# Pharmacology of P2X Receptors and Their Possible Therapeutic Potential in Obesity and Diabetes

**DOI:** 10.3390/ph17101291

**Published:** 2024-09-28

**Authors:** Guillermo A. Cabral-García, José R. Cruz-Muñoz, Eduardo E. Valdez-Morales, Alma Barajas-Espinosa, Andrómeda Liñán-Rico, Raquel Guerrero-Alba

**Affiliations:** 1 Departamento de Fisiología y Farmacología, Centro de Ciencias Básicas, Universidad Autónoma de Aguascalientes, Aguascalientes 20100, Mexico; al64215@edu.uaa.mx (G.A.C.-G.); ray.cruz010893@gmail.com (J.R.C.-M.); eduardo.valdez@edu.uaa.mx (E.E.V.-M.); 2 Consejo Nacional de Humanidades, Ciencias y Tecnologías (CONAHCyT), Ciudad de México 03940, Mexico; andromeda_linan@ucol.mx; 3 Escuela Superior de Huejutla, Universidad Autónoma del Estado de Hidalgo, Huejutla de Reyes 43000, Hidalgo, Mexico; alma_barajas@uaeh.edu.mx; 4 Centro Universitario de Investigaciones Biomédicas, Universidad de Colima, Colima 28045, Mexico

**Keywords:** adenosine 5′-triphosphate, P2X receptors, drugs, obesity, diabetes

## Abstract

The role of P2X ionotropic receptors in the behavior of purinergic signaling on pathophysiological processes has been widely studied. In recent years, the important participation of P2X receptors in physiological and pathological processes, such as energy metabolism, characteristic inflammatory responses of the immune system, and nociceptive activity in response to pain stimuli, has been noted. Here, we explore the molecular characteristics of the P2X receptors and the use of the different agonist and antagonist agents recently described, focusing on their potential as new therapeutic targets in the treatment of diseases with emphasis on obesity, diabetes, and some of the complications derived from these pathologies.

## 1. Introduction

The excessive accumulation of fat in adipose tissue during obesity, as well as the anatomical distribution of these deposits, has a crucial impact on the development of multiple metabolic disorders, including insulin resistance, β-cell dysfunction, prediabetes, and type 2 diabetes (T2D) [1,2]. T2D is a metabolic disorder characterized by metabolic dysregulation of glucose due to loss of insulin sensitization and impaired insulin secretion from pancreatic β cells [3]. Currently, the global incidence of diabetes continues to increase. In 2021, nearly 537 million adults (18–79 years old) were reported to have T2D, and an estimated 783 million people will have T2D in 2045 (International Diabetes Federation). Both obesity and T2D share a chronic systemic inflammatory component that drives the development and progression of insulin resistance [4,5].

Adenosine 5′-triphosphate (ATP) is released from the intracellular medium to the extracellular matrix as autocrine and paracrine mediators in response to metabolic stress and inflammation [6]. The complex network of ligands, enzymes, and receptors that purinergic signaling represents plays an essential role in regulating several physiological processes, including energy metabolism and immune responses [7,8]. In this way, the increase in extracellular ATP is, in turn, a driver of systemic inflammation due to the abnormal activation of purinergic signaling through each of its elements, such as the P2X, P2Y, and adenosine receptors [9]. Currently, several studies detail the importance of the participation of purinergic signaling as well as its elements in the development and progression of multiple diseases, including diabetes [10,11,12].

This review seeks to address some of the most recent knowledge regarding the pharmacological and molecular characteristics of P2X receptors and their role in developing, progressing, and treating comorbidities of obesity and T2D.

## 2. P2X Receptor Pharmacology

P2X receptors (P2XRs) are a family of ligand-gated channels (ionotropic receptors). In mammals, they are widely distributed in the body, and seven subtypes of P2X receptors have been identified (P2X1-P2X7), which are nonselective membrane ion channels (permeable to Na^+^, K^+^, and Ca^2+^) [13].

The main endogenous agonist of P2XRs is ATP. While other endogenous nucleotides such as uridine 5′-triphosphate (UTP), uridine diphosphate (UDP), and UDP-glucose can act as potent P2Y receptor (P2YR) agonists, they have null activity on P2XRs [14]. On the other hand, cytidine 5′-triphosphate (CTP) exerts a slight effect on P2XRs, as its structure is similar to that of ATP; however, because cysteine is a smaller base, CTP is incapable of forming strong bonds with the P2XRs [15].

### 2.1. Molecular Characteristics

In general, functional P2XRs are trimers made up of three monomeric subunits that can assemble as heteromultimers or homomultimers, such as P2X1/5 in some blood vessels, P2X2/3 in ganglion nodosum, P2X2/6 in the brain stem, and P2X4/6 in central nervous system (CNS) neurons [13,16].

**Figure 1:**
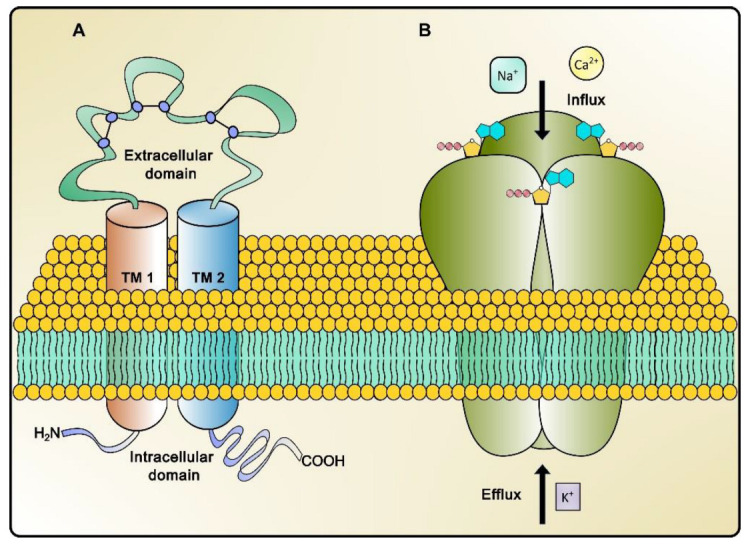
Schematic representations of a monomeric P2X subunit structure (**A**) and a trimeric P2X receptor showing the channel’s pore (**B**).

In turn, each of the seven P2X receptor monomer subtypes consist of an NH2-terminus and two transmembrane domains (TM1 and TM2) separated by a bulky extracellular loop and a COOH-terminal tail, the length of which varies depending on the subtype; both receptor termini (NH2 and COOH) have protein kinase-binding motifs (Figure 1). The TM1 domain is associated with channel activation, while the TM2 domain is part of the ionic pore. The transmembrane loop that separates the TM1 and TM2 domains is formed by multiple disulfide bridges composed of 10 cysteine residues, including ATP-binding sites, competitive antagonists, and metal ion modulation sites [17].

The binding of ATP to the extracellular domain of the P2XR channel leads to changes in the subunits that compose it, thus separating the transmembrane region and causing the opening of the channel [18]. The ability to open the ion channel varies among the subtypes of the P2X family of receptors; P2X2, P2X4, and P2X7 receptors show slower desensitization, while P2X1 and P2X3 receptors have faster desensitization [16]. Three ATP-binding sites have been determined in the transmembrane domain. P2XR structure analysis reveals that ATP phosphates bind to the highly conserved Lys70, Lys72, Arg298, and Asn296 residues, while the adenine base becomes embedded in the extracellular loop, capable of polar (with Lys70 and Thr189) and hydrophobic (with Leu191 and Ile232) interactions, while the ribose ring is recognized by nonpolar residues such as Leu217 [18]. Other agonists, including CTP, have similar binding motifs. The different subtypes of P2X receptors seem to follow the same molecular “rules” for agonist recognition, demonstrating that this mechanism is highly conserved [17,19].

ATP receptor binding induces structural changes, beginning in the binding loop, where the “hardening” of said structure occurs, continuing with the expansion of the lateral regions of the domains where the TM2 α-helices are expanded to the outside in the lower region of the receptor structure and ending with the displacement of the external ends of TM, producing the opening of the pore, emulating an iris [18,20].

Competitive antagonistic binding at P2X receptors is less restrictive compared to that of agonists; for example, the antagonists TNP-ATP and A-317491, by occupying the orthosteric site, bind deeper into the ATP-binding site, conducing to a Y-shaped conformation, which could explain why the opening of the channel does not occur [18].

P2X receptors can be allosterically modulated by ions, including Mg^2+^, Zn^2+^, or Ca^2+^. Moreover, bile acid and lipid steroids, such as phosphatidylinositol polyphosphates (PIPs) that bind to positively charged amino acids at the COOH terminus, inhibit P2X receptor-mediated currents [21]. Karasawa and Kawate (2016) identified that the negative modulation of the pdP2X7 receptor (*Ailuropoda melanoleuca* truncated artificial receptor P2X7) by various antagonist drugs was mediated through binding to an allosteric site other than the ATP-binding site in the extracellular loop [22]; likewise, Wang et al. (2018) determined the structure of hP2X3 (human) in association with AF-219 [5-((2,4-diaminopyrimidin-5-yl)oxy)-4-isopropyl-2-methoxybenzenesulfonamide] and thus differentiated the allosteric binding site from the orthosteric site [23].

### 2.2. P2XR Agonists

As previously described, the ATP-binding site is a highly conserved region amongst the P2X receptors. Despite this, the ATP potential has marked differences between each P2XR subtype. Burnstock and Kennedy (1985) originally defined the agonist potential for P2X receptors through the compounds α, β-methyleneATP (α, β-meATP), 2-methylthio-ATP (2-MeSATP), and ATPγS, which present a profile like ATP, with improved metabolic stability [18]. α, β-meATP has shown great agonist potential on the P2X1 and P2X3, P2X5, P2X6 receptors, also acting on the P2X4/6 and P2X1/5 heteromers and, with a lower potency, on P2X4 and P2X7 [24,25] (Table 1).

### 2.3. P2XR Antagonists

Suramin and PPADS antagonize P2X receptors with low potency and poor receptor subtype selectivity [37]. These characteristics limit their utility as antagonists for P2X receptors, pushing for the development of more potent and selective antagonists for each of the P2X receptor subtypes (Table 2). TNP-ATP is an ATP-derived drug with potent antagonistic potential on some P2XRs such as P2X2 and P2X4 (at nanomolar concentrations) [38]; aurin tricarboxylic acid was demonstrated to have strong nuclease inhibitory potential as well as being a potent noncompetitive blocker of P2X1 and P2X3 receptors [39].

#### 2.3.1. P2X1R Antagonists

P2X1: Antagonistic activity with high potency and selectivity of some salicylamide derivatives have been described [51]. They act as allosteric inhibitors of P2X1 receptors. Some of these derivatives, such as the drug PSB-2014, have only shown partial inhibition of human P2X1 receptors. NF023 (derived from suramin) is a competitive antagonist of P2X1R. However, its selectivity is limited since high concentrations can inhibit P2X3 receptors [38]. NF279 is another suramin derivative with greater antagonist potency towards P2X1R than NF023, registering its activity at nanomolar ranges [52].

#### 2.3.2. P2X2R Antagonists

P2X2: Few selective antagonists to P2X2 receptors have been developed to date. The drug NF770, derived from suramin, presents competitive binding and greater selectivity and potency of P2X2R compared to other P2X receptors [18,53]. Anthraquinone derivatives, PSB-10211 and PSB-1011, are potent antagonists with moderate selectivity to P2X2R. PSB-1011 is a competitive antagonist, with 13 times more potency on P2X2 monomeric receptors [54].

#### 2.3.3. P2X3R Antagonists

P2X3: A wide variety of antagonists specific to P2X3 receptors have been developed with the aim of expanding analgesic and anti-inflammatory treatment options [55]. Drug A-317491 was one of the first selective antagonists described for P2X3 receptors, given its mechanism of action as a competitive antagonist with low oral bioavailability and high binding to plasma proteins [41]. AF-219, a potent allosteric antagonist of P2X3R (also called Gefapixant), has been used in clinical trials to treat chronic cough [43,56]. BAY-181780 is another potent and selective P2X3R antagonist that has been evaluated in clinical trials with positive results [57].

#### 2.3.4. P2X4R Antagonists

P2X4: 5-BDBD and NP-1815-PX, both derived from benzodiazepines, are moderately potent selective allosteric antagonists to P2X4 receptors; however, 5-BDBD denotes low water solubility, while, given the polarity of N.P.- 1815-PX, this drug does not penetrate the central nervous system (CNS) [33,58]. NC-2600, up to now, has been the first selective antagonist to P2X4 receptors that has been evaluated in clinical trials (phase I), demonstrating potent inhibition of human and rodent P2X4R, with no serious side effects reported [59].

#### 2.3.5. P2X5R and P2X6R Antagonists

To date, there are no antagonists that selectively discriminate between P2X5R and the other P2X receptor subtypes [60]. Moreover, P2X6R is mostly retained in the endoplasmic reticulum and fails to incorporate as a homomeric channel in the plasma membrane, although it may do so as a heteromer with P2X2 or P2X4 [61].

#### 2.3.6. P2X7R Antagonists

P2X7: P2X7 receptors have been one of the most widely investigated subtypes for drug development in treating inflammatory diseases [55]. Compound A-740003 (disubstituted cyanoguadinine derivative) is a potent antagonist at high nanomolar ranges, being two times more potent in human P2X7R than rodent P2X7R [46], while A-438079 (disubstituted tetrazolylmethylpyridine derivative) has increased potency [47]. JNJ47965567 is a potent, high-affinity, selective antagonist of human P2X7R and exhibits functional blockade of BzATP-induced IL-1β release [62]. Similarly, it reversed the damage produced in retinal pericytes due to high glucose concentrations in an in vitro model of early diabetic retinopathy [63].

## 3. Link between Obesity and Diabetes

Obesity is characterized by an excess of accumulated body fat accompanied by an inflammatory component generated by a dysfunctional immune response, which triggers changes in the leukocyte count and mediates the ensuing inflammation [64].

Consumption of a high-fat diet (HFD) exacerbates the development of obesity, as well as related metabolic disorders [65,66]. Although obesity has been linked to an imbalance between energy intake and expenditure, the underlying detrimental mechanisms of HFDs are more complicated than the mere concept of energy imbalance [67]. When ingesting an HFD, an alteration in the intestinal microbiota occurs (dysbiosis), typically leading to an increase in *Firmicutes* and a reduction in *Bacteriodetes* phyla [67,68]. Gut dysbiosis compromises the integrity of the epithelial barrier, resulting in an increase in intestinal permeability to bacterial endotoxins such as lipopolysaccharides (LPSs) and free fatty acids (FFAs) [5]. LPSs induce the activation of Toll-like receptors (TLRs) in epithelial and immune cells, promoting the release of proinflammatory mediators and inflammation (referred to as intestinal low-grade inflammation). Proinflammatory cytokines give feedback positively and exacerbate gut permeability [69].

Prior to obesity development, leakage of these microbial and dietary components enters the circulation and triggers inflammatory pathways in multiple organs. Specifically, FFAs and cytokines activate NF-κB/IKKβ signaling in the hypothalamus [70,71], while activated macrophage inflammatory cells (M1) in plasma extravasate to adipose tissue, muscle, and pancreatic islets. Furthermore, during the consumption of an HFD, it is not possible to store excess lipids in adipose tissue, as these are deposited in peripheral tissues such as the liver, skeletal muscle, and blood vessels [72,73]. Thus, HFD-related inflammation makes it difficult for adipocytes to effectively clear circulating FFAs, an essential step for the progression of obesity and the development of other complications [5].

Several factors have been shown to play a critical role in the development of obesity-related insulin resistance, involving ectopic accumulation of fatty acids due to excess unexpended calories, causing hypertrophy and hyperplasia of white adipose tissue (WAT), hypoxia of the tissue itself, alterations in lipid metabolism, aberrant release of FFAs [74], dysregulation of adipokine production (such as elevated levels of the proinflammatory adipokines leptin, resistin, chemerin, progranulin, and monocyte chemoattractant protein-1 (MCP-1) [1,4,75]), and reduced levels of anti-inflammatory adipokines, such as adiponectin and IL-10 [76]. In addition, the large amount of fatty acids stored in hypoxic adipocytes exacerbates some oxidative processes, including lipoperoxidation, that considerably increase reactive oxygen species (ROS) and nitrogen species (RNS), which consequently initiate a localized inflammatory process characterized by increased leptin and TNF-α and decreased adiponectin and IL-10 [77]. Furthermore, changes in adipose tissue promote the recruitment of M1 macrophages in the tissue, which secrete inflammatory factors, including TNF-α, IL-6, IL-1β, IL-8, and nitric oxide [78]. The activation and signaling state of these macrophages are further modulated by the elevated levels of proinflammatory cytokines and excess FFAs, initiating a local positive feedback loop of chronic low-grade inflammation, and in turn, these inflammatory factors are released into circulation and travel to other organs, generating inflammation and endoplasmic reticular stress in them, giving rise to a low-grade systemic inflammatory condition [79].

Systemic inflammation promotes desensitization to insulin and the establishment of insulin resistance, which is defined as a defect in insulin signaling and impaired systemic glucose uptake in adipose tissue, liver, and skeletal muscle [80,81]. Normally, insulin receptor activation promotes the autophosphorylation of several tyrosine residues located in the cytosolic region of the receptor’s β subunit. The autophosphorylated tyrosine residues are then recognized by different adaptor proteins, including members of the insulin receptor substrate 1 and 2 (IRS-1/2) family, which act as adaptor molecules that coordinate the formation of molecular complexes and trigger the insulin signaling cascade for glucose uptake [82]. In contrast, during low-grade systemic inflammation characteristic of obesity, the produced cytokines TNF-α, IL-6, and IL-1β bind to their receptors on the cell membrane of adipocytes and insulin-sensitive tissues (liver, pancreas, and skeletal muscle), triggering an increase in the phosphorylation of serine and threonine residues in IRS-1/2 through the NF-κB/IKKβ and c-Jun N-terminal kinase pathways, which prevents appropriate activation of the insulin receptor signaling pathway and contributes thus to insulin resistance in insulin-sensitive tissues [83,84]. Furthermore, alterations in lipid metabolism in WAT induce elevated levels of triglycerides and FFAs in plasma that accumulate in insulin-sensitive tissues and contribute to insulin resistance in skeletal muscle [85]. Impaired fat oxidation due to mitochondrial dysfunction may contribute to this lipid accumulation. Fatty acid overload in mitochondria generates oxidative products, including ROS, causing the activation of inflammatory kinases and mitochondrial damage that has also been associated with insulin resistance in skeletal muscle [86].

Peripheral insulin resistance causes pancreatic β cells to secrete more insulin, a process known as compensatory hyperinsulinemia. In the long term, this hyperinsulinemia, together with the loss of insulin sensitivity and the increase in the circulation of proinflammatory cytokines, promotes oxidative stress on pancreatic β cells, mitigating the function of the Nrf2 protein (transcription factor responsible for encoding antioxidant enzymes), leading to apoptosis of these cells and sustained hyperglycemia [5,69,87]. The decrease in insulin secretion, coupled with the progression of insulin resistance, can lead to the development of T2D.

## 4. Role P2XRs in Obesity and Diabetes

In the complex pathogenesis of diabetes, particularly T2D, subclinical systemic inflammation plays an important role, and purinergic signaling may be an essential modulator of this inflammatory response. Extracellular ATP levels regulate various physiological processes, including epithelial transport, neurotransmission, and even secretory processes (such as insulin secretion from pancreatic β cells). However, under pathological conditions, extracellular ATP levels are increased, perpetuating the dysregulation of purinergic signaling, which may participate in the inflammatory responses observed in several metabolic disorders. Thus, ATP can act as a DAMP signal in systemic inflammation, activating the ion channels of the P2XRs [88].

The role of P2X receptors in the inflammatory response through immune regulation is widely recognized, and increasing attention is being paid to their expression and function. Monocytes, macrophages, neutrophils, eosinophils, mast cells, T and B lymphocytes, and NK cells express P2X1, P2X4, and P2X7 receptors [89,90]. P2X2 receptors are also expressed in B cells [91] and P2X5 receptors in T lymphocytes [92]. On the other hand, P2X2 and P2X5 receptors have received little attention with regard to the immune system. Therefore, not much information is available on their function. Regarding the P2X function in these cells, it is recognized that P2X1 and P2X4 receptors are involved in T-cell and mast cell activation [93]. The P2X7R is the most widely studied purinergic receptor, and many functions regarding its regulation of the immune response have been demonstrated. For example, the P2X7 receptor can promote inflammatory immune responses and exacerbate inflammatory diseases in the following manners: (1) by activating transcription factors, including NF-κB, nuclear factor of activated T cells (NFAT), and hypoxia-inducible factor 1α (HIF1α); (2) by increasing the expression of the proinflammatory cytokines TNF-α and IL-6 and the chemokines CCL2 and CXCL2 on different immune cells; and (3) by increasing neutrophil recruitment [94]. The P2X7 receptor is also one of the most potent activators of the NLRP3 inflammasome, inducing release of 1L-1β from macrophages and neutrophils [95]. In addition, the P2X7 receptor regulates T lymphocyte survival, differentiation, and activation and is associated with intestinal epithelial barrier dysfunction [96]. Finally, activation of the P2X7 receptor can induce cell death, which leads to the release of damage-associated molecular patterns (DAMPs) and amplifies inflammation [97].

P2XRs play an essential role in the inflammatory response in obesity and T2D (Table 3). More specifically, in humans, there is an active expression of P2X7R in white adipose tissue, which modulates the release of proinflammatory cytokines such as IL-1β, IL-6, and TNF-α, in part, through inflammasome activation [98]. Studies in animal models have suggested that stimulation of P2X7R may have an anti-adipogenic effect, as an increase in body weight, accompanied by adipose hyperplasia and ectopic fat accumulation, have been observed in P2X7R KO mice [99]. Furthermore, studies using a P2X7 knockout mouse also demonstrated that the lack of P2X7 produces the expansion of T follicular helper cells (Tfh) in the Peyer’s patches (PPs) of the small intestine, which leads to increased production of secretory immunoglobulin A (SIgA) and to the abundance of commensals that affect the host’s metabolism, resulting in an obesity phenotype and an alteration of glucose homeostasis [100,101]. On the other hand, another research group found that the reduction in CD36, a membrane glycoprotein that contributes to metabolic disorders such as obesity, was correlated with a decrease in mitochondrial ATP generation and P2X7 expression, suggesting that the suppression of CD36 attenuates adipogenesis via P2X7R [102]. Taken together, these studies suggest that the P2X7 receptor is crucial in regulating lipid metabolism.

In addition to perpetuating systemic inflammation, altering the extracellular concentration of ATP can impact physiological processes in which purinergic signaling is involved. In the pancreas, part of the secretory functions of β cells are regulated through purinergic receptors. In functional studies of β cell secretory activity, ATP has demonstrated a stimulatory effect on insulin secretion through P2X3 receptors [103].

**Table 3:** Anomaly of P2XR in obesity and T2D.

Receptor	Tissue	Δ Gene or Protein Expression	Functional Consequence	References
P2X7	Mice pancreatic β cells	Increased expression of P2X7 in an early stage of obesity and insulin resistance and decreased in later phases of T2D	IL-1Ra secretion and regulation of β cell mass and function	[9,104]
P2X7	Adipose tissue	Increased protein and mRNA expression of P2X7 in metabolic syndrome patients	Modulated the release of inflammatory cytokines and attenuated adipogenesis	[98,102,105]
P2X7	Lymphocyte T	Increased expression of P2X7 in patients with T2D	Associated with an increase in HbA1c and increased fasting plasma glucose level	[106,107]
P2X7	Peripheral blood monocytes	Increased expression of P2X7 in patients with T2D	Associated with increased release of proinflammatory cytokines	[108]
P2X7	Endothelial cells of aortas	No changes in P2X7 expression were reported in a rat model of T2D	Endothelial dysfunction in the aortas by activation of P2X7R	[109]
P2X7	Human retinal pericytes	No changes in P2X7 expression were reported in an in vitro model of early diabetic retinopathy	Regulation of diameter of retinal microvessels and cell apoptosis	[63,110]
P2X4	Hippocampal microglia	Decreased expression of P2X4R in a rat model of T2D	Associated with memory impairment	[111]
P2X4	Satellite glial cells of DRG	Increased expression of P2X4R in a rat model of peripheral diabetic neuropathy	Activation of P2X4R-induced neuropathic mechanical hyperalgesia	[112]
P2X3	DRG cells	Increased expression of P2X3R in diabetic rats	*P2RX3* gene promoter DNA demethylation and enhanced interaction with p65 contribute to diabetic pain hypersensitivity	[113]
P2X2 and P2X7	Colon tissue	Decreased expression of *P2RX2* and *P2RX7* mRNA in a murine T2D model	ND	[114]

DRG, dorsal root ganglia; HbA1c, human glycated hemoglobin; IL-1Ra, interleukin-1 receptor antagonist; ND, not determined.

**Figure 2:**
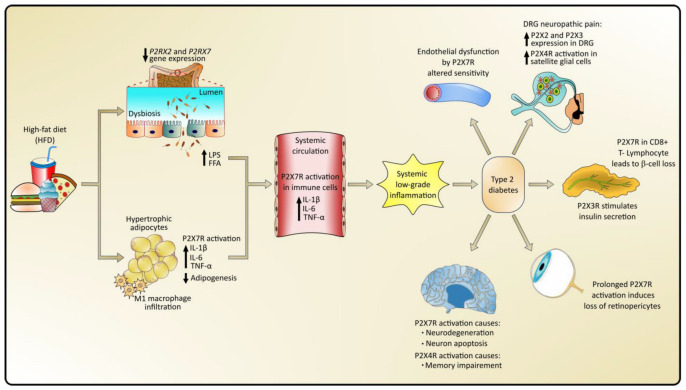
P2XRs might participate in the pathogenesis of obesity and T2D and its complications through mechanisms that involve several tissues and organs, such as the intestine, white adipose tissue, pancreas, blood vessels, and nervous system. High-fat diets induce alterations in the intestinal microbiota and epithelial barrier, causing bacteria and their products, such as lipopolysaccharides (LPSs) and free fatty acids (FFAs), to translocate to the systemic circulation, resulting in low-grade systemic inflammation and alterations in the expression and function of P2XR, which lead to homeostatic alterations in different organs.

Additional studies in β-cells (INS-1E cell line) have shown that P2X7 receptors participate in the regulation of ATP release through pannexin-1, the autocrine stimulation of calcium signaling, and the release of insulin, as well as the regulation of cell proliferation [88,115]. On the other hand, in vitro studies in human and mouse islets reported that in presence of inflammatory conditions and high glucose concentrations, a release of high levels of extracellular ATP is observed, together with an upregulation of P2X7R in CD8+ T lymphocytes, leading to the destruction of the pancreatic islets [107]. Likewise, resistance to β cell loss and hyperglycemia has been reported in P2X7R KO mice treated with streptozotocin (STZ) [116]. According to this evidence, basal concentrations of extracellular ATP can stimulate the proliferation of this type of cells, while higher concentrations stimulate inflammatory processes and cell death (Figure 2).

## 5. P2XRs in the Pathogenesis of Diabetic Complications

The participation of P2X receptors’ signaling in systemic inflammation, characteristic of obesity and diabetes in the gastrointestinal system, has not been fully elucidated. Alteration in purinergic P2X receptors has been linked to gut motility abnormalities associated with these diseases. This seems to be related to dysfunction of the neurons that innervate the enteric nervous system (ENS). Zhang et al. (2019) found that the loss of enteric NOS neurons in diabetic mice is mediated by P2X7 when combined with pannexin-1 to form transmembrane pores that allow macromolecular substances and calcium to permeate the cell membrane [117]. Therefore, symptoms such as constipation, irritable bowel syndrome, and pain, among others, can be attributed to delayed gastro-emptying, as well as abnormal motility, secretion, or absorption in diabetes [118]. Recently, we found that the *P2RX2*, *P2RX7*, *P2RY2*, *A3*, *NTE5*, and *ADA* gene expression is altered in an intestinal low-grade inflammation associated with obesity-induced T2D mouse model, suggesting that these purinergic signaling components might be relevant in the pathophysiology of T2D and could represent potential therapeutic targets in gastrointestinal tract dysfunctional complications related to this disease [114].

A wide range of complications associated with diabetes and the cardiovascular system include hypertension, atherosclerosis, heart disease, microvascular pathologies in various organs, and alterations in blood cells. Purinergic signaling components are altered in the diabetic vascular system, affecting the distribution of receptors in endothelial and smooth muscle cells and culminating in changes in vascular reactivity and vascular smooth muscle function [8,118]. Mahdi et al. (2018) point out that the endothelial dysfunction of the aortas of animals with T2D is partly due to an altered sensitivity of the A1R, P2X7R, and P2Y6R receptors [109]. Zhou et al. (2015, 2017) observed an increase in the vasodilator activity of the agent Uridine Adenosine Tetraphosphate (UP4A) through the P2Y1 receptor in pigs with metabolic disorders, as well as the induction of aortic contraction dependent on the activation of TxSynthase, the T.P. receptor of TXA2, and of P2X1R [119,120].

Diabetic retinopathy (D.R.) is a common complication of diabetes involving capillary abnormalities, often detected in the early stages of diabetes. In retinal cell culture in high glucose concentrations, an increase in the release of ATP has been observed, accompanied by a reduction in its extracellular degradation [121]; likewise, in cultures of retinal neurons and microglia under the same conditions of elevated glucose, it triggers an increment in Ca^2+^ response to P2XR stimulation, possibly leading to the release of neurotransmitters and proinflammatory mediators associated with retinopathy [122].

A link between the activation of the P2X7 receptor and the inflammation characteristic of D.R. has been demonstrated, which is why it has been proposed as a potential therapeutic target [63]. Pericytes have been found to regulate the lumen diameter of retinal microvessels through P2X7R activation [123]. However, prolonged activation of P2X7R can lead to cell apoptosis due to the formation of a macropore that allows the passage of high-molecular-weight molecules in the cytosol, which can contribute to the loss of pericytes in the extracted retinal microvessels from diabetic mice [63,110].

In the diabetic CNS, dysfunction in the neurotransmission systems has been detected, implicating the purinergic system in memory impairment and neurodegeneration [8]. Additional studies have found alterations in ATP signaling, as well as adenosine accumulation in the cerebral cortex of diabetic rats, an upregulation of A2AR and P2X7R, as well as a downregulation of A1R, whereas others found a decrease in nucleotide hydrolysis and an increase in adenosine deaminase activity in the brains of hyperglycemic zebrafish [12,124]. Thus, high levels of ATP can promote the activation of P2X7R, triggering the entry of Ca^2+^ and generating harmful effects leading to the activation of apoptotic pathways in neurons [125,126].

Diabetic neuropathy is characterized by atrophy and loss of nerve fibers; in STZ-induced murine diabetes, a reduction in cutaneous innervation, together with a decrease in the expression of P2X3R in the skin of the footpad, was observed [127]. Likewise, there is an increase in the expression of P2X2R and P2X3R, together with a higher current density in dorsal root ganglia (DRG) of diabetic rats and mice post-STZ treatment, whereas pain hypersensitivity in the hind paw was significantly decreased when treated with Suramine or A-317491 [113,128]. Likewise, there is evidence of P2X4R activation in microglial cells within the CNS participating in developing neuropathic pain in diabetic peripheral neuropathy [112]. Teixeira et al. (2019) found functional expression of the P2X4 receptor in DRG satellite glial cells (SGC), whose activation is essential for the development of mechanical hyperalgesia induced by diabetic neuropathy.

In the future, research on P2X receptors may provide new insights into the mechanisms underlying the development and progression of the diabetes-induced complications, including the ones mentioned above. For example, studies might explore the expression and activity of different P2X receptor subtypes in adipose tissue, liver, muscle, and pancreatic beta cells, all of which are involved in glucose metabolism. More research is required to understand the effects of P2XR modulation on glucose homeostasis, insulin sensitivity, and pancreatic β cell function in patients with T2D. Likewise, insights into the interaction of T2D and other comorbidities, such as cardiovascular disease, diabetic retinopathy, and chronic kidney disease, open new strategies to prevent or treat these complications.

Medicinal chemistry has made substantial advancements by providing more specific therapeutic chemicals that specifically target P2X receptors [18]. Several of these drugs have undergone assessment in preclinical investigations or progressed to clinical trials, demonstrating both effectiveness and tolerability (Table 4). As an example, Gefapixant, a selective antagonist of the P2X3 receptor, has been utilized as a treatment for chronic cough [56]. JNJ54175446, a selective antagonist of the P2X7 receptor, shows promise as a potential treatment for major depressive illness [129]. These chemicals broaden the potential for testing as treatments for additional disorders, including diabetes and obesity.

Overall, the field of purinergic signaling research concerning T2D, especially involving P2X receptors, together with advances in medical chemistry, appears to have a bright future and has the potential to unlock new insights into the underlying mechanisms of the disease. Moreover, it may facilitate the development of new therapies to improve the prognosis of patients who suffer from this chronic illness.

## Figures and Tables

**Table 1:** List of EC_50_ values of P2X receptor agonists commonly used as pharmacological probes.

Receptor	Compound	EC_50_ Value [μM]	References
P2X1	ATP	0.056 (h); 0.1–0.3 (r)	[26,27,28]
	2-MeS-ATP	0.054 (h); 0.1 (r)	
	ATPγS	2.3 (h); 0.59 (r)	
	α,β-MeATP	0.2 (h); 3.2 (r)	
	β,γ-MeATP	2.0 (h); 8.7 (r)	
	BzATP	0.002 (h); 24.2 (r)	
	AP_4_A ^1^	0.182 (h)	
	AP_6_A	0.6–0.72 (r)	
	CTP	35.1 (r)	
	ADP	30 (h)	
P2X2	ATP	2.0–8.0 (h); 3.7 (r)	[27,29]
	2-MeATP	1.0 (h); 1.5 (r)	
	ATPγS	1.5 (r)	
	BzATP	5.5 (h)	
	ADP	100 (h)	
	AP_4_A	15 (h)	
P2X3	ATP	1.0 (h); 1.0 (r)	[26,27]
	2-MeS-ATP	0.350 (h); 0.3 (r)	
	ATPγS	0.690 (h)	
	α,β-MeATP	0.740 (h); 1.0 (r)	
	β,γ-MeATP	9.2 (r)	
	BzATP	5.5 (h)	
	AP_4_A ^1^	0.80 (r); 15.0 (h)	
	AP_5_A^1^	1.3 (r)	
	AP_6_A	1.6 (r)	
P2X4	ATP	0.74 (h); 1.0–10 (r); 0.35 (m)	[26,30,31]
	2-MeS-ATP	7.4–100 (r)	
	ATPγS	2.3 (r)	
	β,γ-MeATP	3–10 (r)	
	BzATP	0.515 (h); 300 (r); 2.9 (m)	
	AP_4_A ^1^	1.0 (h)	
P2X5	ATP	0.44–15.4 (r)	[28,30]
	2-MeS-ATP	0.44–20 (r)	
	ATPγS	0.29–9.3 (r)	
	α,β-MeATP	1.1–100 (r)	
	β,γ-MeATP	11.8 (r)	
	BzATP	1.3 (r); 40 (h)	
	AP_3_A ^1^	5.4 (r)	
	ADP	1.8 (r)	
P2X6	ATP	12 (h); 1.0–10 (r)	[30]
	2-MeS-ATP	9 (h); 0.6 (r)	
	ATPγS	1.3 (r)	
	BzATP	25 (r)	
	ADP	11 (r)	
P2X7	ATP	780 (h); 100 (r)	[26,31,32]
	2-MeS-ATP	178 (h); 2000 – 4000 (m); 10 (r)	
	ATPγS	138 (h)	
	α,β-MeATP	>300 (r)	
	BzATP	52 (h); 5.0–500 (r)	
	ADP	>300 (r)	

^1^ Partial agonist; (h) human, (r) rat, and (m) mouse P2X receptor in a heterologous expression system as 1321 N cells [33], Xenopus laevis oocytes [27,28,34,35,36], and HEK cells [30].

**Table 2:** IC_50_values of P2X receptor antagonists commonly used as pharmacological probes.

Receptor	Compound	IC_50_ Value [μM]	References
P2X1	Suramin	1.0 (h); 1.0–1.7 (r)	[26,28,39,40]
	PPADS	1.0 (h); 0.09–0.12 (r)	
	Reactive Blue 2	2.3 (r)	
	PPNDS	14 (r)	
	TNP-ATP	0.006 (h); 0.001 (r)	
	IP_5_1	0.001–0.003 (r)	
	MRS2159	1.15 (h); 0.009 (r)	
	MRS2220	10.2 (r)	
	MRS2219	5.9 (r)	
	ATA	0.008 (r)	
P2X2	Suramin	10.4 (r)	[26,35]
	PPADS	1.2 (r)	
	NF023	>50 (r)	
	Reactive Blue 2	0.360 (r)	
	TNP-ATP	2 (h)	
	MRS 2179	>10 (r)	
	IP51	>30 (r)	
	MRS 2220	>100 (r)	
	NF770	0.019 (h)	
	PSB-10211	0.09 (r)	
	PSB-1011	0.08 (r)	
P2X3	Suramin	14.9 (h); 0.006–3.0 (r)	[26,40,41,42,43]
	PPADS	1.7 (h); 0.005–1.0 (r)	
	TNP-ATP	0.9 (h)	
	MRS2179	13 (r)	
	IP_5_I	2.8 (r)	
	MRS2220	58.3 (r)	
	A-317491	0.02–0.1 (h)	
	AF219-MK7264	0.03 (h)	
	RO-4 or AF-235	0.008 (h, r)	
	BAY1817080	0.008 (h)	
	BLU- 5937	0.025 (h)	
P2X4	Suramin	100 (h); 500 (r)	[33,44,45]
	PPADS	30 (h); 200 (r)	
	NF023	>100 (h)	
	Reactive Blue 2	128 (h)	
	TNP-ATP	15.2 (h)	
	KN-62	>100 (h)	
	MRS2220	>100 (r)	
	5-BDBD	0.35–0.5 (h); 3.5 (r); 2.5 (m)	
	NP-1815-PX	0.26 (h)	
	PSB-12054	0.19 (h)	
	PSB-12062	1.4 (h)	
	BX430	0.78 (h)	
	BAY-1797	0.11–0.23 (h, m, r)	
	PSB-15417	0.022–0.037 (r); 0.087 (m)	
P2X5	Suramin	1.0–4.0 (r)	[28]
	PPADS	0.20–2 (r)	
	TNP-ATP	0.45 (r)	
	Reactive Blue 2	18.5 (r)	
	IP5I	<30 (r)	
P2X7	Suramin	300 (h); 300 (r)	[26,39,46,47,48,49,50]
	PPADS	62.2 (h); 50 (r)	
	MRS2179	>30 (h)	
	A-438079	0.933 (h)	
	A-804598	0.010–0.021 (h, r, m)	
	A-740003	0.04–0.069 (h)	
	A-839977	0.02 (h); 0.042 (r); 0.150 (m)	
	AZ11645373	0.007–0.1(h)	
	CE224,543	0.002–0.013 (h)	
	GSK1482160	0.003 (h)	
	AZ9056	0.012 (h)	
	AZ10606120	0.0014–0.23 (h)	
	JNJ47965567	0.005–0.011 (h)	
	JNJ54232334	0.003 (h); 0.032 (r)	
	JNJ54140515	0.079 (r)	
	JNJ54175446	0.003 (h)	
	JNJ54173717	0.0016 (h)	
	JNJ42253442	0.020 (h)	
	JNJ64413739	0.015 (h)	
	GW791343	8.9 (h)	

Abbreviations: (h) human, (r) rat, and (m) mouse P2X receptor.

**Table 4:** Drugs in clinical trials targeting P2X receptors.

ID No./Drug Name	Target Receptor	Disease/Symptoms	Phase	Trial Status/Sponsor Name
NCT02502097/ Gefapixant (AF-219/MK-7269)	P2X3R antagonist	Idiopathic pulmonary fibrosis with persistent cough [57]	III	Completed/Afferent Pharmaceuticals, Inc., a subsidiary of Merck & Co., Inc. (Rahway, NJ, USA)
NCT04614246/ BAY-1817080	P2X3R antagonist	Endometriosis with pelvic pain [130]	II	Terminated/Bayer (Leverkusen, Germany)
NCT04545580/ BAY-1817080	P2X3R antagonist	Overactive bladder [131]	II	Completed/Bayer (Leverkusen, Germany)
NCT04693195/ BLU-5937	P2X3R antagonist	Chronic pruritus associated with atopic dermatitis [132]	II	Completed/Bellus Health Inc.—a GSK company (London, UK)
NCT04110054/ S-600918	P2X3R antagonist	Adults with refractory chronic cough [133]	II	Completed/Shionogi Inc. (Shuo-ku, Japan)
NCT05305183/ Minodronate	P2X2/3R antagonist	Osteoporosis; back pain in patients with osteoporosis [134]	III	Unknown/Shandong New Time Pharmaceutical Co., Ltd. (Linyi, China)
NC-2600	P2X4R antagonist	Neuropathic pain [135]	I	Unknown/Nippon Chemiphar Co., Ltd. (Chiyoda-ku, Japan)
AK-1780	P2X7R antagonist	Diabetic peripheral neuropathy/chronic pain [136]	II	Unknown/Eli Lilly and Company (Indianapolis, IN, USA)
NCT00628095/ CE-224535	P2X7R antagonist	Rheumatoid arthritis in patients who have not totally improved with methotrexate [137]	II	Completed/Pfizer (New York, NY, USA)
NCT00418782/ CE-224535	P2X7R antagonist	Osteoarthritis/chronic pain [138]	II	Terminated (lack of efficacy)/Pfizer (New York, NY, USA)
NCT00849134/ GSK1482160	P2X7R antagonist	Inflammatory pain [139]	I	Completed/GlaxoSmithKline (London, UK)
NCT04116606/ JNJ-54175446	P2X7R antagonist	Major depressive disorder [129]	II	Unknown/CCTU-Core (Cambridge, UK)
NCT05328297/ JNJ-55308942	P2X7R antagonist	Bipolar depression [140]	II	Completed/Janssen Pharmaceutica N.V., Belgium (Beerse, Belgium)
NCT05620576/ LY3857210	P2XR antagonist	Diabetic peripheral neuropathic pain	II	Completed/Eli Lilly and Company (Indianapolis, IN, USA)

## Data Availability

Data are contained within the article.
